# Assessment of Perceived Effort Through On-Field Hydration Monitoring: A Case Analysis

**DOI:** 10.3390/life14111447

**Published:** 2024-11-08

**Authors:** Alexander Bertuccioli, Roberto Cannataro, Davide Sisti, Giordano Bruno Zonzini, Massimiliano Cazzaniga, Marco Cardinali, Francesco Di Pierro, Aurora Gregoretti, Nicola Zerbinati, Mariarosaria Matera, Ilaria Cavecchia, Chiara Maria Palazzi

**Affiliations:** 1 Department of Biomolecular Sciences, University of Urbino Carlo Bo, 61029 Urbino, Italy; alexander.bertuccioli@uniurb.it (A.B.); davide.sisti@uniurb.it (D.S.); giordano.zonzini@uniurb.it (G.B.Z.); marco.cardinali@uniurb.it (M.C.); 2 Microbiota International Clinical Society, 10123 Torino, Italy; maxcazzaniga66@gmail.com (M.C.); f.dipierro@vellejaresearch.com (F.D.P.); auroragregoretti@gmail.com (A.G.); jajamatera74@gmail.com (M.M.); ilaria.cavecchia@gmail.com (I.C.); pchiaramaria@gmail.com (C.M.P.); 3 Galascreen Laboratories, University of Calabria, 87036 Rende, Italy; 4 Research Division, Dynamical Business and Science Society, DBSS International SAS, 110311 Bogota, Italy; 5 Scientific & Research Department, Velleja Research, 20125 Milano, Italy; 6 Department of Internal Medicine, Infermi Hospital, AUSL Romagna, 47921 Rimini, Italy; 7 Department of Medicine and Surgery, University of Insurbia, 21100 Varese, Italy; nicola.zerbinati@uninsubria.it

**Keywords:** endurance, electrolytes, salivary osmolarity

## Abstract

This case report examines the correlation between hydration, weight variation, and perceived effort in a 43-year-old amateur athlete during a self-supported 81.5 km crossing of Death Valley, completed over 3 days with significant elevation changes. Studies have shown that a body mass loss greater than 2–3% can lead to an increased perception of effort and a decline in performance. Specifically, during passive and active heat exposures, the average body mass loss was found to be 1.4 ± 0.3% and 4.1 ± 0.7%, respectively. Salivary osmolarity has demonstrated a sensitivity of 86% and specificity of 91% in diagnosing dehydration of ≥ 2%, suggesting its potential as a non-invasive indicator of hydration status. The subject monitored their own body weight, hydration (via salivary osmolarity), and perceived effort using a rate of perceived exertion (RPE) scale. Nutritional intake included isocaloric meals and nutritional bars, and hydration was managed using water and a hydroelectrolytic solution. Key bioimpedance parameters were measured to assess body composition and hydration status. A progressive decrease in body weight correlated with an increase in perceived effort (RPE score) and salivary osmolarity. Resistance (Rx) remained stable, while reactance (Xc) showed a biphasic trend and was inversely correlated with the sodium/potassium ratio (NAK). There were significant linear correlations between perceived effort and both weight loss and salivary osmolarity, indicating that salivary osmolarity is a potential early predictor of these changes. The findings highlight a linear correlation between weight loss, perceived effort, and salivary osmolarity, suggesting that monitoring salivary osmolarity would be useful for the field assessment of hydration and exertion. Further research with larger populations is necessary to validate these observations.

## 1. Introduction

During long-duration events, especially under extreme temperatures, fluid intake is essential to compensate for losses. A fluid loss greater than 2–3% of the body mass (BM) is typically associated with an increased perception of effort, a rise in core body temperature, and a consequent decline in performance [1]. Body water losses greater than 2% of the BM are defined as hypohydration. Changes in hydration status can be assessed using a variety of body measurements, including the evaluation of urine color [2] or plasma osmolality. Blood osmolality is considered by many as the gold standard for assessing hydration status [3,4]. Sodium levels, in addition to plasma osmolality, have also been used to assess an individual’s hydration status, as both increase proportionally to the degree of dehydration. The sensation of thirst has also been suggested as a perceptual surrogate marker for dehydration; however, because thirst is stimulated by significant dehydration, individuals may already be dehydrated by the time they feel thirsty. This makes it a less precise and accurate parameter compared to, for example, urinary biomarkers, such as urine specific gravity (USG) and urine osmolality (UOsm), which vary in response to dehydration. On the other hand, urine color can also serve as a valid and practical indicator [2]. Due to the low variability in their measurement, changes in BM provide the most sensitive and straightforward measure for determining acute changes in body fluids. Researchers have investigated new biomarkers, such as saliva, sweat, and even tears, as potential biological samples for assessing hydration status. Salivary osmolality and saliva flow have proven to be promising indicators of hydration [5]. It is obviously important to establish a personalized fluid replenishment plan to avoid the risk of developing hyponatremia. Furthermore, dehydration can increase the risk of gastrointestinal discomfort [6] and other health and performance risks including acute kidney injury (AKI) [7,8], hyponatremia (EAH), and muscle cramps (EAMC) [9].

The hypothesis underlying this study is that salivary osmolality (SOSM), monitored directly in the field, may serve as a non-invasive indicator of hydration status and may correlate with other physiological parameters, such as body weight variation and perceived effort level. Based on this hypothesis, we describe the case of an amateur athlete undertaking a self-supported 81.5 km crossing of Death Valley, completed in three days in total self-sufficiency.

Upon monitoring the performance and health condition of the participating athlete with the aim of managing and optimizing future performance, an interesting correlation between SOSM and hydration monitored in the field using bioelectrical impedance vector analysis (BIVA) emerged, leading to the drafting of this work. This strategy, if developed, could prove useful in improving hydration management in the field.

## 2. Materials and Methods

**In this case** report, we describe the observed correlation between field-assessed hydration and salivary osmolarity (SOSM), a non-invasive, real-time, quantitative, and cost-effective method for evaluating hydration levels [10]; weight variation; and the level of perceived effort observed in a 43-year-old Caucasian amateur athlete. These were analyzed before, during, and after a self-supported crossing of Death Valley. The 81.5 km route was completed through alternating phases of light running and walking, facing an elevation gain of 1206 m and a descent of 2815 m. The total duration was 3 days, and the participant completed the crossing completely self-sufficiently, carrying all the necessary supplies in a backpack with an initial weight of 22 kg and a final weight of 14 kg (due to the consumption of water and provisions). This study was conducted according to the principles stated in the Declaration of Helsinki and was approved by the Ethics Committee for Human Experimentation of Urbino University Carlo Bo (no. of approval 29_2020).

### 2.1. Subject

The subject is a 43-year-old Caucasian male, weighing 97.3 kg and standing 183 cm tall, with a BMI of 29.3 and a fat mass of 15%, as evaluated using a BIA101 BIVA PRO bioimpedance analyzer (Akern, Florence, Italy) [11,12,13,14,15,16,17], with the data processed using Bodygram Dashboard^®^ software version 3.0.27 (Akern, Florence, Italy) [14,15,16,17], and BIATRODES electrodes (Akern, Florence, Italy) [14,15,16,17]. Prior to the BIVA, the subject was positioned supine on a non-conductive surface for at least 5 min to ensure an even distribution of fluids. The lower limbs were abducted at a 45° angle from the body’s midline, and the upper limbs were abducted at a 30° angle from the trunk, ensuring there was no contact between the lower limbs, upper limbs, trunk, or any potentially conductive object. The skin was cleansed with alcohol at the electrode application points (two injectors and two detectors), which were placed on the back of the right hand at the radioulnar joint and the metacarpophalangeal joint of the third finger for the upper section, and on the back of the foot with one at the tibiotarsal joint and the other at the metatarsophalangeal joint of the third toe for the lower section, maintaining a distance of 5 cm between each electrode [11,12,13,14,15,16,17].

The subject has over 10 years of experience in running and trail running, and in the new context of a self-supported crossing, he independently and voluntarily decided to monitor data related to his body weight, hydration, and body temperature, while also monitoring his perceived exertion with the aim of studying and optimizing his performance, subsequently providing written consent for the data’s use in a completely anonymous manner for the analysis of the findings. Analyzing the recent competitive profile reveals that the subject has participated in approximately 54 competitive events over the past three years, ranging between 20 and 70 km. From a training standpoint, his weekly workload varies depending on the period, ranging from 40 to 75 km of running, divided between slow endurance runs, technique work, specific running drills, etc. In terms of strength training, on average, two sessions per week lasting about 60–75 min are carried out, including resistant strength work and core stabilization, along with corrective exercises for postural imbalances, joint mobility, and general flexibility. Reviewing the subject’s medical history reveals that his cardiometabolic profile is normal, and medical clearance for competitive physical activity has been granted in accordance with Italian regulations. No significant medical events have occurred, except for a left ankle subluxation that happened approximately 13 months prior to this assessment, which occasionally requires appropriate medical treatment for recurring tendon issues and peritendinitis. At the time of the assessment, these conditions were not present. This case presentation was redacted according to the Care guidelines [18].

### 2.2. Nutrition, Supplementation, and Hydration

During the crossing, meals were concentrated in the two moments when the group was camped, breakfast and dinner, where it was possible to prepare hot drinks (tea or coffee) and rehydrate the freeze-dried foods used. These foods consisted of isocaloric mixtures providing an average of 500 kCal, 60 g of carbohydrates, 20 g of fats, and 20 g of proteins (Tactical Foodpack, Hilden, Germany) [19]. During the activity, nutritional bars were consumed every 2.5 h, providing 204 kCal, 19 g of carbohydrates, 7 g of fats, and 15 g of proteins (Balanced bar—Keforma, Acquaviva, San Marino Republic) [20]. Because hydration had to be carefully self-managed, 750 mL of water and 750 mL of an unflavored hydroelectrolytic solution enriched with glucose (KeSali Hypotonic—Keforma, Acquaviva, San Marino Republic) [21] were consumed daily. This provided a daily caloric intake of about 1850 kcal. To further optimize fluid loss through sweating, the athlete used a full outfit made entirely of FIR fabric, enabling more efficient management of body heat and promoting recovery and supercompensation [22], which was created considering the body mapping of regional sweat distribution [23].

Following his usual supplementation protocol, the athlete took the following nutraceuticals:

Breakfast:

- A mixture of cocoa flavanols and hawthorn (Biovaleoxifull—Biovale, Serravalle, San Marino Republic) [24,25,26];
- A mixture of coenzyme Q10 and B vitamins (DDM-Chinone—Pharmextracta, Pontenure, Italy) [27];
- Curcumin in phytosomal form (Algocur—Pharmextracta, Pontenure, Italy) [28,29];
- Clostridium butyricum CBM588 (Butirrisan—Pharmextracta, Pontenure, Italy) [30];
- A mixture of cherry flavonoids and Tanacetum parthenium (Freedoms bar—Keforma, Aquaviva, San Marino Republic) [31].

Dinner:

- Curcumin in phytosomal form (Algocur—Pharmextracta, Pontenure, Italy) [28,29];
- Clostridium butyricum CBM588 (Butirrisan—Pharmextracta, Pontenure, Italy) [30];
- A mixture of cherry flavonoids and Tanacetum parthenium (Freedoms bar—Keforma, Aquaviva, San Marino Republic) [31];
- A mixture of hydrolyzed collagen and elastin with vitamin C (DDM-Matrice—Pharmextracta, Pontenure, Italy) [32].

### 2.3. Monitoring Tools and Protocol

At the beginning and end of each day, all measurements were taken (except for height, which was assessed only before departure) using the equipment that the subject carried along with the equipment in the backpack. Weight was measured using a miniaturized scale, whose accuracy was previously compared in the clinic with a SECA 700 Eye-Level Beam mechanical column scale (SECA North America, Medical Measurement Systems and Scales, 13601 Benson Avenue, Chino, CA 91710, USA) [33]. Height was assessed before departure using the stadiometer included in the SECA scale. The bioimpedance parameters resistance (Rx) and reactance (Xc) (and the derived estimate of the sodium/potassium ratio, NaK) were measured using a BIA101 BIVA PRO bioimpedance analyzer (Akern, Florence, Italy) [11,12,13,14,15,16,17], with the data processed using Bodygram Dashboard^®^ software (Akern, Florence, Italy) [14,15,16,17], and BIATRODES electrodes (Akern, Florence, Italy) [14,15,16,17], strictly following the manufacturer’s protocol. NaK is an indirectly obtained parameter that reflects the ratio between extracellular sodium ions and intracellular potassium ions, and is consequently related to intra- and extracellular fluid levels. 

Hydration status was monitored using a point-of-care MX3 (MX3 Diagnostics Austin, TX 78704, USA) device, capable of assessing salivary osmolarity, an element predictive of hydration status, strictly following the manufacturer’s protocol [34,35,36,37]. These devices were selected for their technical characteristics, easy portability, and light weight. Perceived effort was assessed using a rate of perceived exertion (RPE) scale that included values from 1 to 10, where 1 corresponded to no perceived effort and 10 to maximum perceived effort. Once the journey was completed, the data were processed using Excel 365 (Microsoft, Redmond, WA, USA) [37] and SPSS 22.0 (IBM, Armonk, NY, USA) [37].

### 2.4. Characteristics of the Route

The crossing took place on terrain that included mountain passages, reaching a maximum altitude of 2181 m and a minimum of −15 m below sea level, with most of the route being off marked trails, crossing natural territories with full respect for the local flora and fauna. The recorded temperatures ranged from a minimum of 11 °C to a maximum of 35 °C, with significant variations depending on the section of the route.

### 2.5. Statistical Analysis

Descriptive statistics are reported using linear and 3rd degree polynomial regression; the goodness of fit was quantified using determination coefficients (r2); R2 indicates the proportion of deviance explained with respect to the total deviance. The regression functions used are reported. Correlation matrices were obtained using Pearson coefficients. All the analyses were performed using Excel 365 [37].

## 3. Results

As is reported in Table 1 some data (weight, salivary osmolarity, and perceived effort) show a linear and consistent trend, demonstrating a good correlation, as will be described later. Resistance shows a slight decrease, indicating relative stability, while other measures (Xc and NAK) exhibit rather peculiar dynamics.

**Figure 1:**
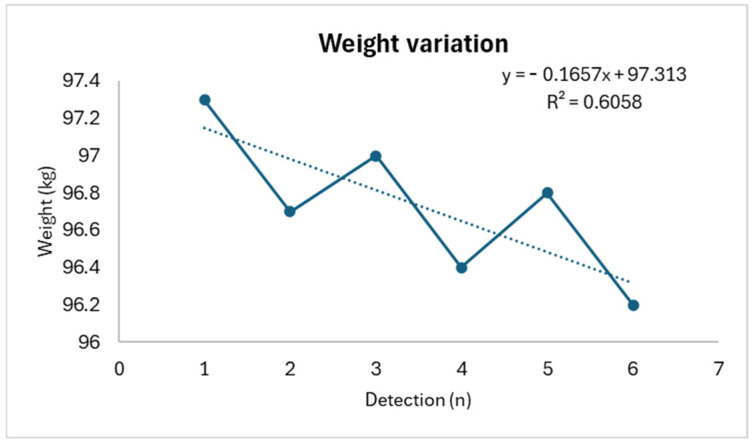
Weight trend.

**Figure 2:**
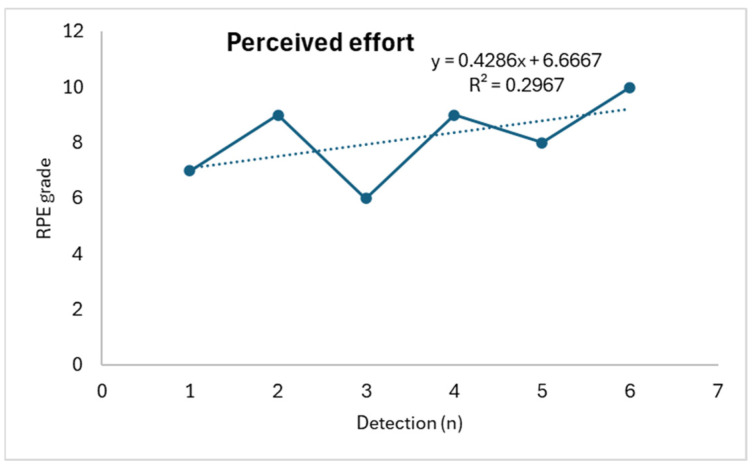
Perceived effort trend.

**Figure 3:**
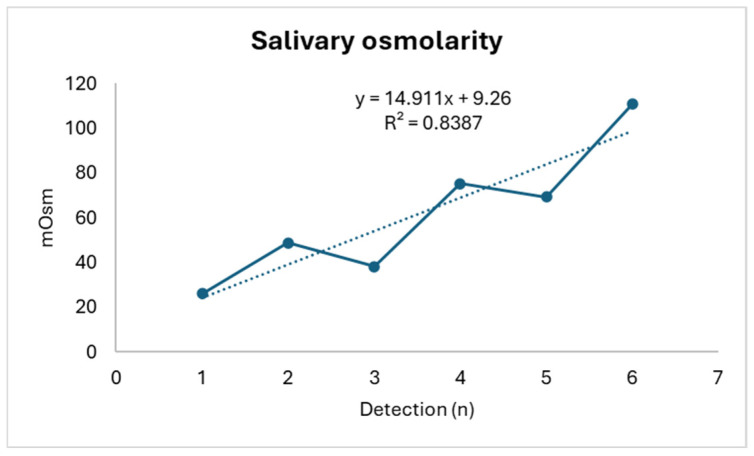
Salivary osmolarity trend.

In detail, weight shows a progressive decrease (R^2^: 0.6058) concurrently with a progressive increase in perceived effort (R^2^: 0.2967) (with an increase in the RPE score) and salivary osmolarity (R^2^: 0.8387), as shown in Figure 1, Figure 2 and Figure 3.

**Figure 4:**
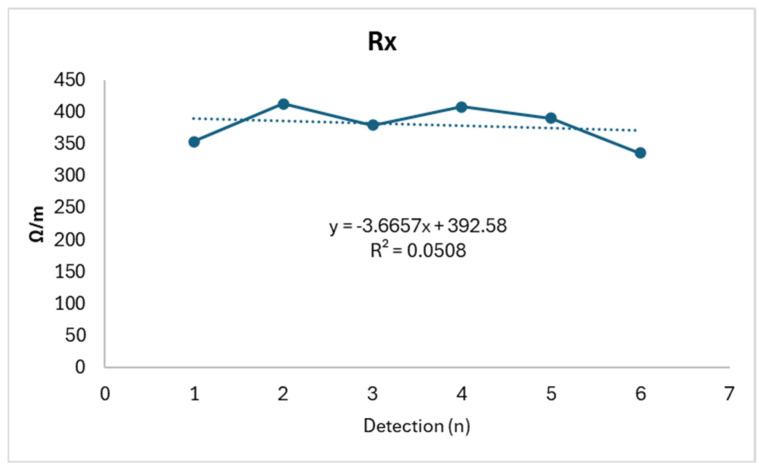
Rx trend.

**Figure 5:**
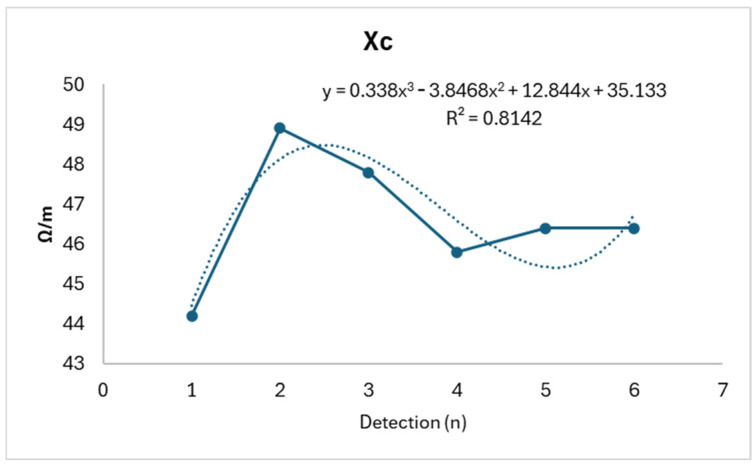
Xc trend.

**Figure 6:**
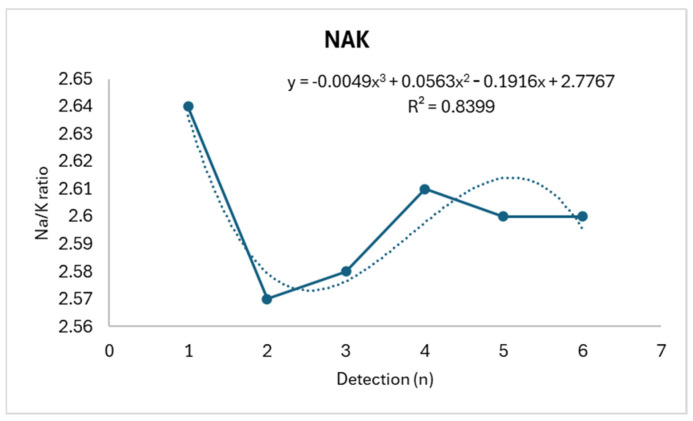
NAK trend.

Rx shows a slight reduction consistent with relative stability (R^2^: 0.0508), while Xc displays a biphasic trend, showing an initial increase followed by a subsequent decrease (R^2^: 0.8142). Meanwhile, NAK exhibits an almost opposite trend (R^2^: 0.8399), as shown in Figure 4, Figure 5 and Figure 6.

Upon analyzing the linear correlations between the various parameters considered, a strong negative linear correlation between perceived effort and weight (r: −0.864) and a strong positive linear correlation between perceived effort and salivary osmolarity (r: 0.812) emerge. NAK shows a strong linear correlation with Xc (r: −0.990), as shown in Table 2.

## 4. Discussion

The aim of this case report was to evaluate whether salivary osmolality (SOSM) can serve as a non-invasive indicator of hydration status, correlating with other physiological parameters, such as body weight variation and perceived effort level.

Many factors contribute to an individual’s performance capacity: training, nutrition, gut microbiota health [38,39], and hydration management [20]. During physical exercise, especially in hot environments, many people do not adequately replace the fluids lost through sweat, which leads to an involuntary reduction in body water (dehydration). To monitor dehydration, blood biomarkers that indicate an increased blood concentration are often used. However, salivary osmolality appears to be a promising indicator for assessing hydration status [3].

The analysis of the parameters considered highlights two specific scenarios. The first shows a linear correlation, likely predictable, between the reduction in weight, presumably due to fluid loss, and the increase in perceived effort. In this context, a linear correlation between the reduction in weight and increase in salivary osmolarity is observed, as is that between the increase in perceived effort and increase in salivary osmolarity. This potentially suggests that salivary osmolarity could serve as an early predictive element for detecting weight loss and the onset of perceived effort. This element could be particularly useful for field monitoring and self-monitoring, where the size and weight of the equipment and the speed at which the technique is executed are particularly relevant.

The biphasic trend in reactance, concurrently with the inverse dynamics of NAK, highlights a likely acute adaptation situation, consistent with what is found in subjects undergoing hemodialysis, where similar variations are inversely correlated with the volume of fluids removed and the relative variations in intra- and extracellular ionic dynamics [22,40]. The consideration of these aspects could allow for planning appropriate hydration strategies before and in the early stages of a performance, to improve hydrosaline stability.

### 4.1. The Importance and Significance of These Observations

Analyzing performance in this context is crucial to prevent issues such as acute kidney injuries (AKIs) reported in people participating in marathons and trail running. Current knowledge indicates that these issues are correlated with inadequate hydration management and the use of NSAIDs (non-steroidal anti-inflammatory drugs) before or during ultra-distance runs [8]. This correlation is attributed not only to an increased likelihood of developing potentially ischemic kidney damage, such as acute tubular necrosis, but also to risks like exertional rhabdomyolysis, exercise-associated hyponatremia, and gastrointestinal symptoms. These interconnected conditions can further elevate the risk of AKI, underscoring the necessity for developing strategies to mitigate this risk [7]. Furthermore, the wide variability in current hydration strategies often proves inadequate for optimal athlete management [41]. 

An effective hydration strategy is recognized as essential for optimizing performance, particularly in long-distance races [42]. Moreover, it is generally deemed safe and effective for elite athletes to exceed current hydration guidelines during ultra-endurance activities [43]. This raises new questions regarding performance dynamics that necessitate the development of rapid analytical methods and in-depth investigations. Additionally, better hydration management could facilitate improved prevention strategies for electrolyte imbalances, particularly sodium levels, which may contribute to muscle cramps (EAMC) and exercise-associated hyponatremia (EAH) [9]. This phenomenon should not be overlooked, as it reportedly affects an average of 8% of individuals participating in runs over 40 km [44].

Several methods have been proposed for monitoring hydration status in the field, including the analysis of salivary osmolarity and urine color, and indirect assessment of vital signs and subjective thirst sensations. However, these methods exhibit numerous limitations, primarily due to a lack of sensitivity, reliability, and accuracy. This highlights the necessity for developing new approaches, methods, and correction techniques for the existing data. Validation against complex and invasive methods (e.g., neutron activation analysis and stable isotope dilution) could help to address these limitations. Both bioimpedance vector analysis (BIVA) and salivary osmolarity analysis currently have several constraints for routine use, necessitating further research on larger samples across various environmental conditions [45]. The observed correlation between SOSM and bioimpedance parameters may serve as a preliminary indication for future studies aimed at developing more effective hydration assessment methods.

Studies on changes in hydration status during both passive and active heat exposure have revealed that the average body mass loss was 1.4 ± 0.3% during passive exposure and 4.1 ± 0.7% during active exposure. Notably, salivary osmolarity has proven to be an effective indicator for diagnosing dehydration of ≥ 2% during exercise, with a sensitivity of 86% and specificity of 91% [37]. In a protocol involving intense physical exercise, it was observed that a body mass loss of 3% resulted in a significant increase in salivary osmolarity among all participants, highlighting a positive correlation between dehydration and salivary osmolarity regardless of sex [46]. However, it is essential to consider the differences in the secretion of salivary components between males and females [47]. In elderly hypertensive patients undergoing diuretic therapy, studies have shown a strong correlation between urine specific gravity and salivary osmolarity when analyzing morning samples. Salivary osmolarity correlated well with urine specific gravity measurements obtained using a refractometer, yielding a correlation coefficient (r) of 0.78 (*p* < 0.0001). Furthermore, salivary osmolarity demonstrated excellent predictive ability, with area under the curve (AUC) values of 0.94 and 0.90 for urine specific gravity thresholds of ≥1.025 and ≥1.030, respectively. The optimal salivary osmolarity threshold for detecting dehydration was identified as 93 mOsm, with a sensitivity of 78.6% and specificity of 91.1%. These findings support the use of salivary osmolarity as a non-invasive biomarker for hydration assessment in this population [10,35]. Additionally, it is important to note that salivary osmolarity is significantly altered after a brief rinse with water, necessitating a wait of fifteen minutes before taking valid measurements. This timing is crucial to avoid artifact data and ensure an accurate assessment of hydration status [48]. Summarizing numerous physiological aspects related to the subject’s condition [49,50,51,52,53], biological sex [54,55,56], and the type of activity performed [57,58] can make interpreting data from a salivary osmolarity analysis challenging [59]. 

### 4.2. Limitations

While this study provides valuable insights, several limitations should be acknowledged:

(1) Single-Subject Design: The case report is based on a single subject, which limits the generalizability of the findings. Future studies should include larger sample sizes to validate these observations across diverse populations;
(2) Self-Reported Measures: Perceived effort was assessed using a visual analog scale, which is subjective and may be influenced by various factors. Objective measures of exertion could complement these findings;
(3) Short Duration: The study was conducted over a brief period (3 days), and longer-term monitoring would provide more comprehensive insights into the relationship between hydration and perceived effort;
(4) Environmental Factors: The unique conditions of Death Valley may not be representative of other environments, and the findings may differ in different climates or terrains.

## 5. Conclusions

In this case report, we present the variations in acute parameters observed in a subject who self-sufficiently completed a 81.5 km route through alternating phases of light running and walking in Death Valley, facing an elevation gain of 1206 m and a descent of 2815 m, using a specific integration protocol and specific technical clothing. The results demonstrate a linear correlation between weight loss, an increase in the perceived effort score, and an increase in salivary osmolarity. This suggests that salivary osmolarity could be monitored in the field as a predictor of body weight loss and an increase in perceived exertion. Further studies on large populations and under controlled conditions, overcoming the limitations previously discussed, will be necessary to verify the validity of these observations at the level of specific populations.

## Figures and Tables

**Table 1:** Trends in the main parameters evaluated over the three days, pre- and post-activity.

Observation		Weight (kg)	Salivary Osmolarity (mOsm)	Perceived Effort RPE	Rx (Ω/m)	Xc (Ω/m)	NAK
Day 1	Pre-activity	97.3	26.1	7	353.5	44.2	2.64
	Post-activity	96.7	48.9	9	412.7	48.9	2.57
Day 2	Pre-activity	97	38.2	6	378.8	47.8	2.58
	Post-activity	96.4	75.4	9	407.7	45.8	2.61
Day 3	Pre-activity	96.8	69.3	8	390.3	46.4	2.6
	Post-activity	96.2	110.8	10	335.5	46.4	2.6

**Table 2:** Correlation matrix (Pearson) relating to the parameters examined.

Variable	RPE	Weight	Rx	Xc	Salivary Osm	NaK
RPE	1 *	−0.864 *	0.002	0.093	0.812 *	−0.111
Weight	−0.864 *	1 *	−0.020	−0.234	−0.927 *	0.307
Rx	0.002	−0.020	1 *	0.494	−0.254	−0.467
Xc	0.093	−0.234	0.494	1 *	0.022	−0.990 *
Salivary osm	0.812 *	−0.927 *	−0.254	0.022	1 *	−0.116
NAK	−0.111	0.307	−0.467	−0.990 *	−0.116	1 *

* Values are different from 0 at the alpha = 0.05 significance level.

## Data Availability

The original contributions presented in the study are included in the article, further inquiries can be directed to the corresponding author.
